# Aerodynamic Mechanisms and Flow Physics of Bioinspired Slotted Wingtips

**DOI:** 10.1093/icb/icag036

**Published:** 2026-05-05

**Authors:** Hannah Wiswell, Aimy Wissa

**Affiliations:** Mechanical and Aerospace Engineering, Princeton University, 41 Olden Street, NJ 08544, USA; Mechanical and Aerospace Engineering, Princeton University, 41 Olden Street, NJ 08544, USA

## Abstract

Bird wings contain several feather groups, some of which contribute to their aerodynamic performance during flight. One of these groups is the emarginated primary feathers, which exhibit slots, bending, and twisting in flight. Slotted wingtips vary in morphology across the avian clade, raising questions about the relationship between their form and function, particularly with regard to their aerodynamic role. This study expands the current understanding of the functional morphology of slotted wingtips by systematically studying slots, bending, and twist using various engineered wingtip configurations: one that captures the slotting only, another that has both slotting and bending, and a third that combines slots, bending, and twist. Force, moment, and PIV data acquired during wind tunnel testing reveal that the bioinspired wingtips have both global and local aerodynamic effects. The wingtips’ global aerodynamic effect is to delay spanwise stall propagation, thereby altering the lift distribution over the wing. Local aerodynamic effects include the reduction of aerodynamic load over the wingtips as well as changes to the separated shear layer location and the breakdown of tip vorticity. The results show that while global aerodynamic effects are universal across all configurations, local effects are sensitive to wingtip design. Nonetheless, both the global and local effects enable structural resilience and effective roll and yaw control authority. These results demonstrate that the emarginated primary feathers may have multiple aerodynamic functions, offering new insights into their role in bird flight and showcasing their potential as flow- and flight-control devices for engineered aerial vehicles.

## Introduction

**Fig. 1:**
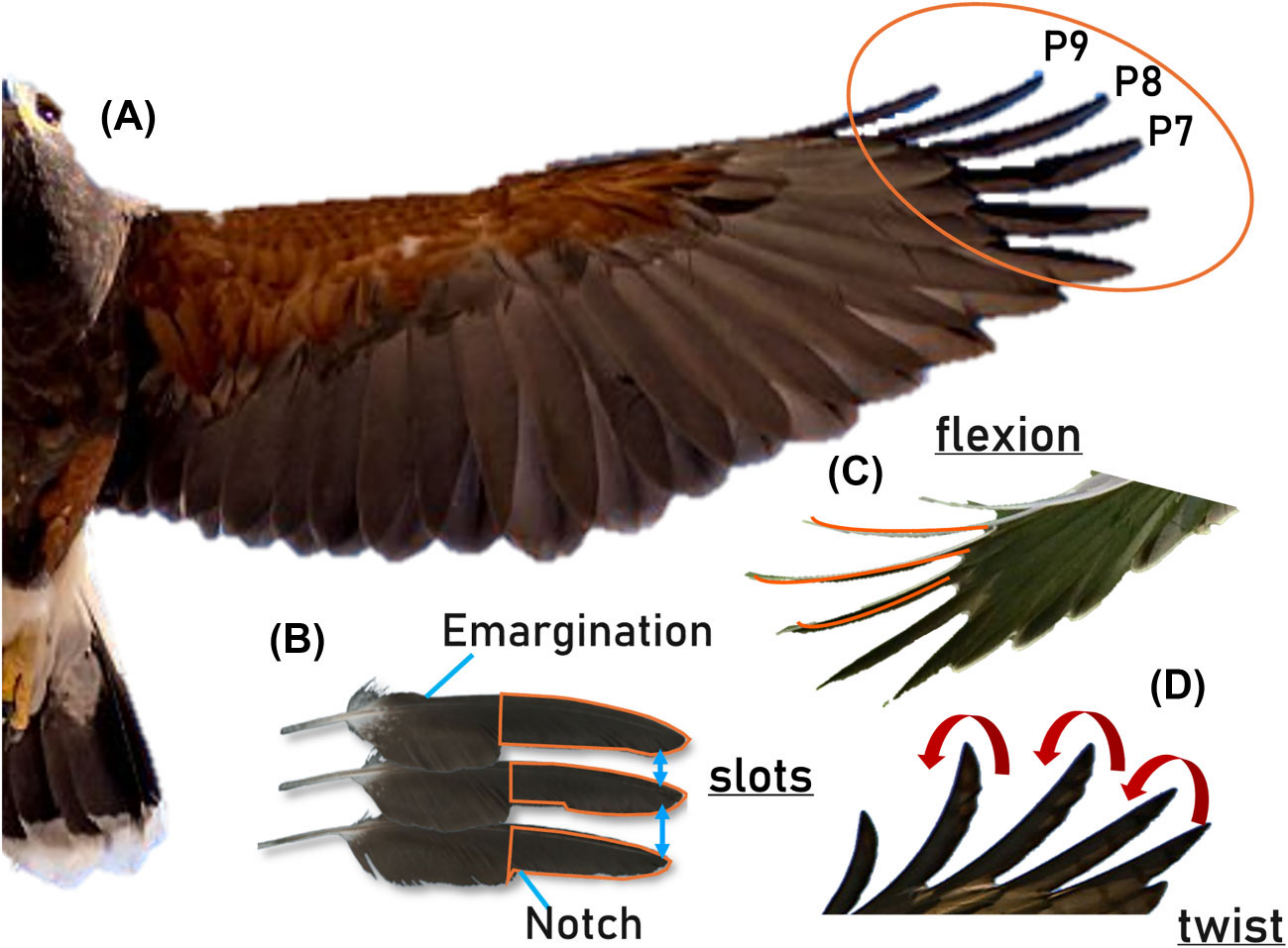
(A) The semi-span of a Harris’s hawk wing with the primary feathers circled and the three primaries of interest, P7, P8, and P9, highlighted. The three feather degrees of freedom (DoFs) are isolated to the following: (B) Slots, formed by an emargination and a notch. Here, the solid outlines at the emarginated section of primaries 7, 8, and 9 show the tracings used to create the slotted wingtip configuration. (C) Flexion, indicated by solid curved lines. (D) Twist, indicated by curved arrows. Image credit: Pixabay.

Bird wings contain spatially distributed feather groups. Several studies have suggested that these feathers may employ various flow-control mechanisms, either passive or active (Graham 1932; Carruthers et al. 2007; Othman et al. 2023). The entire repertoire of flow-control mechanisms associated with one particular feather group, the emarginated primary feathers, which form slotted wingtips, remains unclear. Slotted wingtips, as they will be referred to for the remainder of this study, are formed due to abrupt decreases in width on the anterior and posterior vanes of the primary feathers, resulting in leading edge emarginations and trailing edge notches (Fig. 1A) (Videler 2006). The morphology of slotted wingtips is broadly expressed across the avian clade, which has prompted questions about their possible function in flight and beyond (van Oorschot Klaassen et al. 2017).

One of the first observations of slotted wingtips in flight was made by Robert Graham (1932). Graham noted that beyond the presence of slots (Fig. 1B), the slotted wingtips incorporated additional degrees of freedom (DoFs) by bending and twisting in flight (Fig. 1C and D). Later observations of slotted wingtip deployment were made in 1959, when Newman noted that black vultures kept their slotted wingtips open while soaring and closed them while gliding (Newman 1958). From these observations, Newman hypothesized that these feathers may aid in controlling flow separation at high angles of attack. Since these initial observations, several studies in both the biology and engineering fields have focused on relating the form of slotted wingtips to their aerodynamic function.

In biology, the effect of slotted wingtips on drag has been investigated by flying a freely gliding Harris’s hawk in a wind tunnel before and after clipping its tip feathers (Tucker 1995). With the removal of the slotted wingtips, there was an increase in drag. This was further explored by comparing the performance of an unslotted wingtip to a wingtip with three tandem balsa wood airfoils and a wingtip with Harris’s hawk primary feathers (Tucker 1993). Force measurements and flow visualization of the wake with bubbles in a wind tunnel revealed that slotted wingtips reduced drag and spread vorticity both horizontally and vertically. Following this, KleinHeerenbrink et al. flew a jackdaw in a wind tunnel and used stereo particle image velocimetry (PIV) to analyze flow physics in the wake and on the wingtip region of the wing in gliding and flapping flight (KleinHeerenbrink et al. 2017). Multiple cores of tip vortices were found to originate from the separated primary feathers, demonstrating the slotted wingtips’ ability to spread vorticity horizontally and vertically, possibly reducing induced drag.

Spillman was among the first to study slotted wingtips in an engineering context (Spillman 1978). The study varied the number and geometric parameters of slotted cambered airfoils attached to a wing and tested them in a wind tunnel and in free flight using a Morane-Saulnier Paris aircraft. Spillman found that the wingtips increased the effective aspect ratio by over 40$\%$, reducing induced drag and thus increasing flight efficiency. Following this, Lynch et al. varied the gap size percentage between engineered slotted wingtips, defined as the chordwise distance between the wingtips divided by the base wing chord length, and their planarity (Lynch et al. 2018). With a gap size of 20$\%$ of the wing chord and nonplanar wingtips, induced drag was reduced at a cost of increased parasitic drag. Lee and Wissa extended this study by incorporating incidence angle using rigid wingtips formed from the traced planform of Harris’s hawk primaries (Lee and Wissa 2020). They found that while the aerodynamic efficiency, defined as the ratio of lift to drag, of a wing with slotted wingtips was lower than that of a wing with an unslotted wingtip extension, the wingtips generated lift close to that of the wingtip extension. Additionally, they hypothesized that asymmetric actuation of the wingtips could achieve roll control over a wide range of angles of attack due to a difference in lift between two wings if each is equipped with a different wingtip configuration. More recently, Midmer and Brücker sought to explore the effect of flexible wingtip distribution across the wing chord on flow physics in the wake (Midmer and Brücker 2024). They created flexible wingtip extensions matching the Cauchy number, the ratio between inertial and elastic force, of the peregrine falcon and conducted wind tunnel tests at two angles of attack. Time-resolved PIV revealed that when the slotted wingtips vary in span, tip vorticity is spread in the spanwise and vertical directions, but this feature disappears when the slotted wingtips have the same span length. Thus, the primary hypotheses of previous biological and engineering studies on slotted wingtips are that they reduce induced drag and spread tip vorticity, and there have been a few discussions about their potential to control flow separation and enable roll control. Prior biological studies either used full bird wings or natural feathers, in which all feather features are fully coupled, and prior engineering studies isolated only a single parameter, such as wingtip shape or flexibility. Moreover, most prior studies focus on the wake region, ignoring spanwise flow physics.

The effects of the observed DoFs, namely slotting due to emargination and notching, bending due to flexibility, and twist due to material anisotropy, are difficult to isolate and study in birds. Thus, in this study, we develop and evaluate an engineering analogy that incorporates biologically relevant DoFs, namely slots, bending, and twist. The analogy is evaluated using wind tunnel experiments that yield force- and time-resolved PIV measurements of the flow over the wing section and in the wake at various spanwise locations. The results provide new insights into the role of slotted wingtips in flight and demonstrate their potential as bioinspired flow-control devices for improved aircraft performance.

## Methods

### Experimental setup

**Fig. 2:**
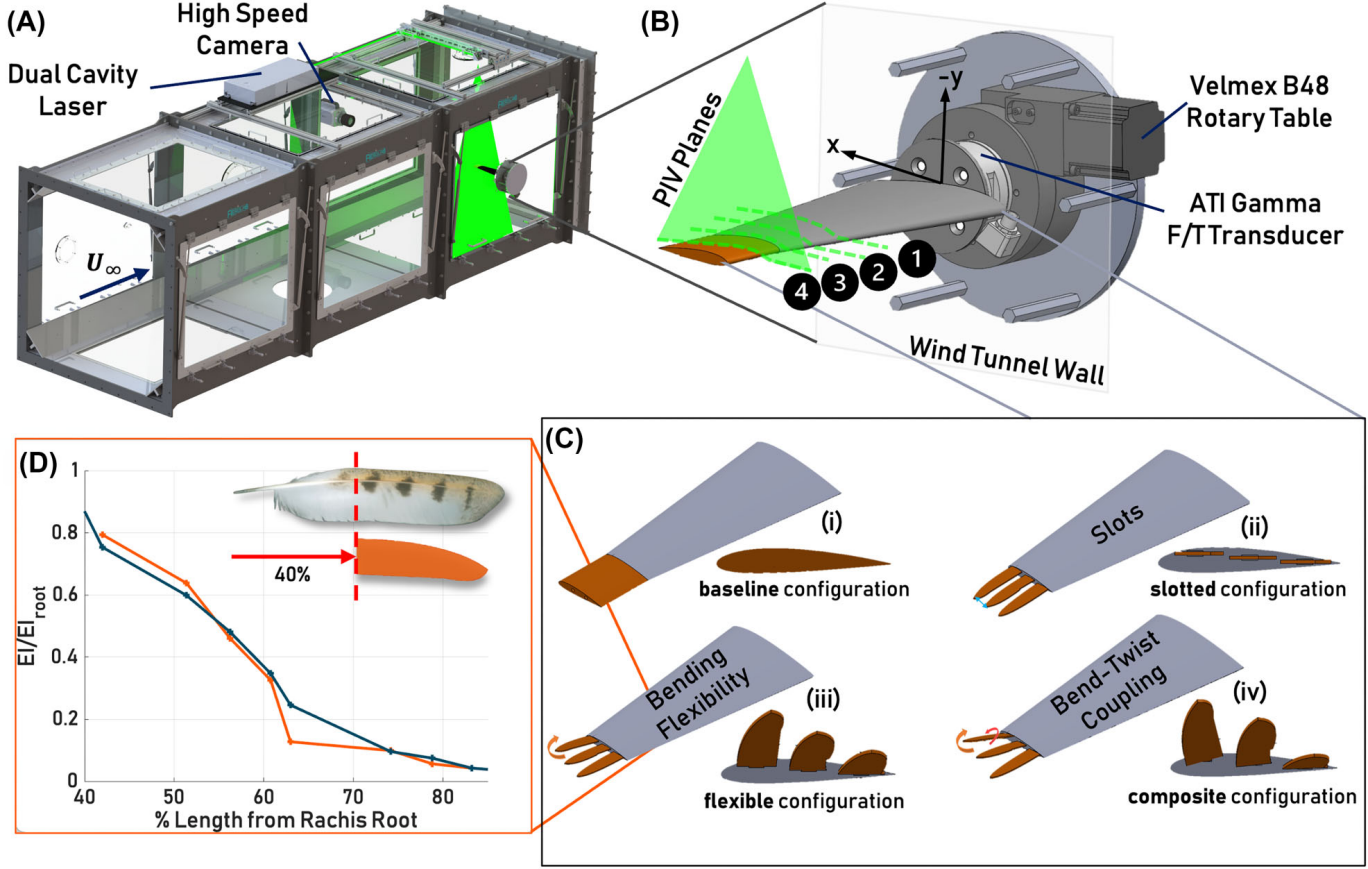
An overview of the experimental setup used for evaluation. (A) A render of the test sections of the subsonic wind tunnel facility at Princeton University which shows the wing attached in the third test section. (B) The wing’s attachment to the six-axis ATI Gamma force/torque transducer and Velmex B48 rotary table, with the four PIV plane locations shown on the suction side of the wing. The axis of measurement for the ATI Gamma is shown with arrows corresponding to x and -y. (C) Each wingtip configuration: baseline (i), slotted (ii), flexible (iii), and composite (iv), with associated testing metrics denoted on the suction surfaces of the wingtip configurations. (D) Normalized flexural stiffness $EI$ as a function of the percentage length from the rachis root, where 40$\%$ represents the root of the manufactured wingtips. The orange line corresponds to the flexible wingtips, and the dark teal corresponds to data from the fifth primary of a barn owl (*Tyto alba*) (adapted from Bachmann et al. 2012).

Wind tunnel experiments were conducted in the closed-loop subsonic wind tunnel facility at Princeton University, which is shown in Fig. 2A. A detailed description of the wind tunnel specifications can be found in Breuer et al. (2022). The experiment was conducted in the third test section of the tunnel, which measures 1.2 m by 1.2 m in cross-section and is 1.4 m long. Tests were conducted at a bird flight-relevant Reynolds number $Re = 2\times10^{ 5}$ with a freestream velocity $U_\infty = 35\,\,$m/s and characteristic length *c* equal to the mean chord of the wing. The tunnel has a turbulence intensity of 0.03$\%$ of the freestream velocity. During the experiments, the angle of attack $\alpha$ was varied between 0$^\circ$ and 40$^\circ$ to capture both pre-stall and post-stall regimes using a Velmex B48 rotary table with a precision of 0.0125$^\circ$. At each angle of attack, force and moment measurements were acquired for a 5-second duration at a sampling rate of 1 kHz using a six-axis ATI Gamma force/torque transducer (Fig. 2B). The ATI Gamma has a range of 0-32 N and a resolution of 1/160 N for the *x* and *y* force channels. Additionally, the torque channels in the *x* and *y* directions have a range of 0-2 Nm and a resolution of 1/2000 Nm. Three trials were conducted for each configuration to acquire an average and standard deviation $\sigma$.

Lift *L* and drag *D* were calculated by translating the aerodynamic forces measured in the wing’s body frame with $\alpha$ as described in Equation (1), where $F_x$ and $F_y$ are the forces measured by the force transducer in the *x* and *y* directions, respectively. These forces were then normalized into the lift $C_L$ and drag $C_D$ coefficients using the dynamic pressure and respective planform area of each test configuration, as described in Equation (2), where $\rho$ is the density of air at room temperature and *S* is the planform area. The rolling moment $M_L$ and the yawing moment $M_N$ were measured in addition to the forces and correspond to the outputs of the ATI transducer’s $-T_x$ and $T_y$ channels, respectively.


(1) \begin{eqnarray*}
L = -F_y\cos \alpha -F_x\sin \alpha ,\,\,\,\,\,\,\,\,D=-F_y\sin \alpha +F_x\cos \alpha\\
\end{eqnarray*}

(2) \begin{eqnarray*}
C_L = \frac{L}{\frac{1}{2}\rho U_{\infty }^2 S},\,\,\,\,\,\,\,\,C_D = \frac{D}{\frac{1}{2}\rho U_{\infty }^2 S}
\end{eqnarray*}

Time-resolved two-dimensional (*x* and *y* planes) and two-component (*u* and *v* velocities) (2D/2C) PIV measurements were acquired to analyze the spanwise flow physics. The PIV setup uses a Photonics DMX Nd:YLF 527 nm dual-cavity high-repetition laser with an acquisition rate of 1 kHz. The laser beam passes through a series of 90° mirrors and is adjusted with converging and diverging lenses to ensure a uniform beam shape and a sheet thickness below 2 mm. To illuminate the suction side and regions fore and aft of the wing, the beam passes through a –10 mm cylindrical lens and fans out. The pressure side of the wing is not included in these measurements because the laser shadow from the suction side obscures it. The tunnel is seeded with Di-Ethyl-Hexyl-Sebacat particles with an average diameter of 1 *μ*m for several minutes before data acquisition. A Photron Nova R5 high-speed CMOS camera with a 9 MP resolution is used to acquire image pairs at 1 kHz. Image post-processing is performed in LaVision’s DaVis software, where time-averaged flow fields are retrieved by averaging over 1000 frames collected over 1 s, with first and second passes of $128\times 128$ and $16\times 16$, respectively, with a $50\%$ overlap. The time increment between each image pair was $40\mu s$. PIV flow fields were obtained for four streamwise planes of interest, at approximately $0.83b$ (Plane 4, located approximately at the wingtip mid-span), $0.78b$ (Plane 3, located at the tip of the base wing), $0.73b$ (Plane 2, located 0.015 m inboard of the base wing), and $0.70b$ (Plane 1, located 0.022 m inboard of the base wing), where *b* is the wing semi-span, as shown in Fig. 2B.

### Wing and wingtip design

The test section for this study is a wing-wingtip system with a total semi-span *b* of 0.3 m and a mean chord *c* of 0.9 m. Each wing-wingtip system consists of the base wing and a wingtip configuration. The base wing has a taper ratio $\frac{c_t}{c_r} = 0.5$, where $c_t$ and $c_r$ are the base wing’s tip and root chords, respectively. This was chosen to minimize any abrupt changes in geometry from the base wing to each wingtip attachment. An NACA 2414 airfoil cross-section was chosen because of its extensive aerodynamic characterization. During the experiments, the root of the base wing was connected to the force/torque transducer and the rotary table to measure the resulting aerodynamic forces and moments and change $\alpha$, respectively.

Four different wingtip configurations were tested, all of which have a span of 0.2*b*. The first configuration, referred to as the baseline, involves a wingtip extension that has a chord length equal to $c_t$ (Fig. 2(C)(i)). The planform area of this wing-wingtip system was approximately 0.024 m$^2$. The second wingtip configuration, referred to as the slotted configuration, is designed to isolate the effect of slots (Fig. 2(C)(ii)). This configuration features three wingtips designed from tracings of the emarginated sections of primaries 7, 8, and 9 of an adult Harris’s hawk (Fig. 1B), as these are the primaries with the most pronounced emargination for that species. The slotted wingtips have a NACA 2414 cross-section with a mean chord length of approximately 0.18$c_t$ and are made from Rigid 10K resin. The wingtips’ bases have an equal slot spacing of 0.3$c_t$. Given their material properties, the slotted wingtips remain rigid and do not deflect under wind-induced loads (supplemental video 1). The planform area of this wing-wingtip system was approximately 0.022 m$^2$.

The remaining two configurations have the same wing-wingtip planform area as the slotted configuration, but differ in their material properties and cross-sectional geometry. The third wingtip configuration is the flexible configuration, which is designed to enable bending flexibility similar to that exhibited in bird flight (Fig. 2(C)(iii), supplemental video 2). The flexible wingtips are 3D printed using TPU 95A. The thickness of the flexible wingtip is chosen to approximate the flexural rigidity of bird primary feathers. Existing feather flexural stiffness data was used to guide the design of the flexible wingtips, specifically data characterizing the flexural stiffness $EI$ of barn owl feathers at different locations along the fifth primary shaft as described in Bachmann et al. (2012). Using the Young’s modulus *E* of TPU 95A, the cross-sectional thickness of the flexible wingtips and the corresponding second moment of area were designed to match the $EI$ of the biological primary feather (i.e., $EI_{flex.wingtips}=EI_{owl}$). Figure 2D shows the normalized flexural stiffness as a function of the percentage length from the rachis root, with 40$\%$ representing the root of the manufactured wingtips.

The final configuration is referred to as the composite configuration (Fig. 2(C)(iv)). This configuration is designed to enable bend-twist coupling, incorporating the final DoF: twist. Each composite wingtip has a cross-sectional thickness equal to the average cross-sectional thickness of its corresponding flexible wingtip. For example, composite P7’s cross-sectional thickness is equal to flexible P7’s average cross-sectional thickness. The composite wingtips are made with PLA fibers enclosed by a TPU95A matrix with a fiber volume fraction of 0.47. The fiber orientation angle and the stacking sequence were chosen to maximize the bend-twist coupling of the composite wingtips while limiting fabrication complexity, as described in Gustafson et al. (2019). The composite wingtips used in this study have a fiber orientation angle of 30$^\circ$ and a stacking sequence of [30]$_2$. When subjected to a wind-induced load, the composite wingtips passively twist due to aerodynamically induced bending (supplemental video 3). Their resulting incidence angles when subjected to the wind-induced load are not necessarily accurate to those expected from real primaries, as the composites were designed to maximize the bend-twist coupling of engineered wingtips. The composite wingtips have the same flexural stiffness throughout their span, calculated as a weighted average of *E* for TPU95A and PLA multiplied by their respective second moments of area, *I*. The slotted, flexible, and composite configurations are collectively referred to as the bioinspired wingtip configurations in the results.

## Results

**Fig. 3:**
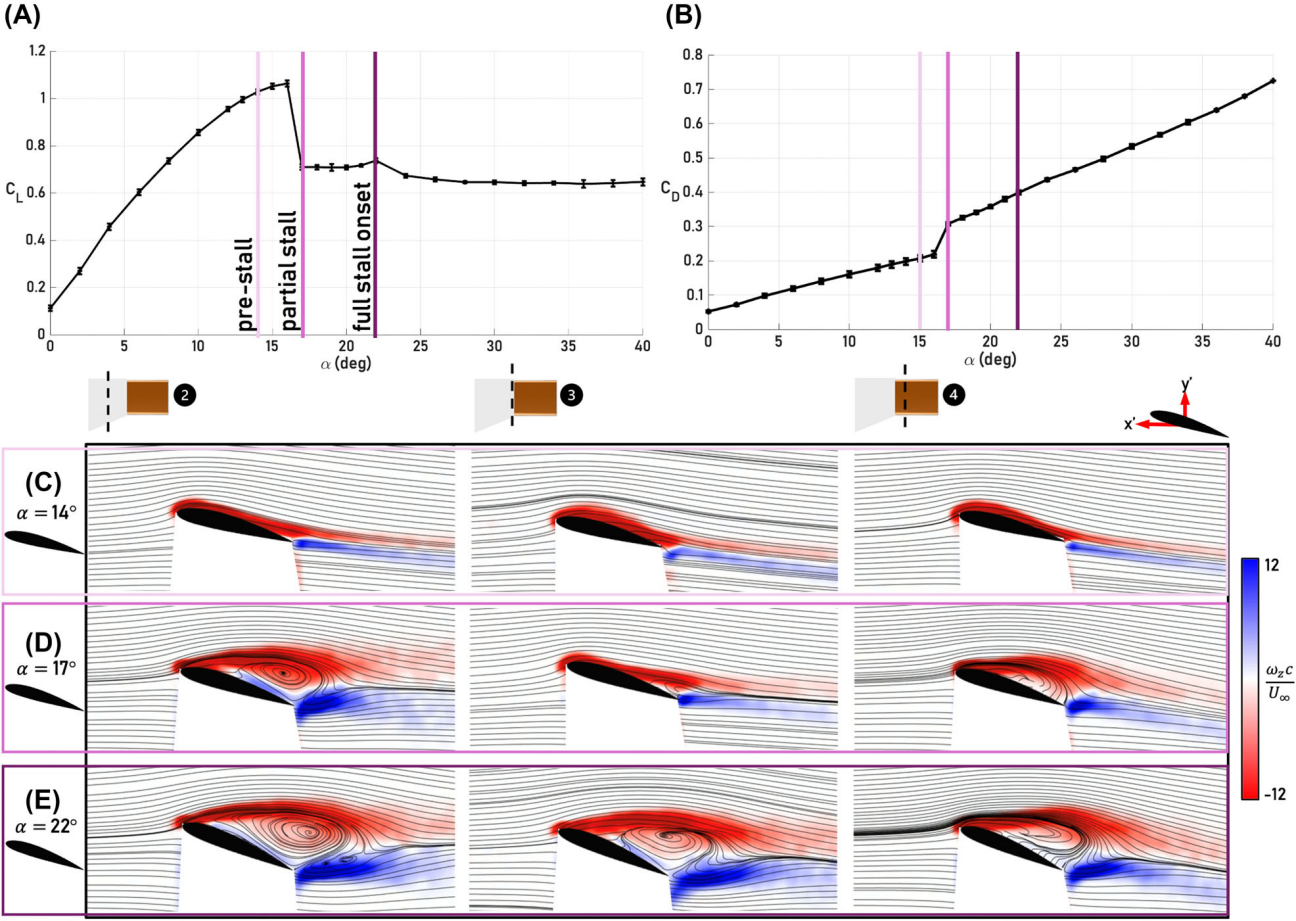
Aerodynamic performance and flow field results for the baseline configuration. (A) Mean coefficient of lift $C_L$ versus angle of attack $\alpha$ for the baseline with vertical lines denoting the locations of pre-stall (14$^\circ$), partial stall (17$^\circ$), and full stall onset (22$^\circ$). Error bars here represent $\pm \sigma$. (B) Mean coefficient of drag $C_D$ versus $\alpha$ for the baseline with the same vertical lines showing pre-, partial, and full stall onset locations with error bars representing $\pm \sigma$. (C) Flow fields showing the spanwise vorticity $\omega _z$ normalized by the mean chord *c* and freestream velocity $U_\infty$ at $\alpha = 14^\circ$ for Planes 2, 3, and 4 (left to right), where uniformly colored streamlines represent the velocity magnitude. The PIV-relevant axis with the wing frame-of-reference is shown on the airfoil in the top right, denoted by arrows for *x*’ and *y*’. (D) As (C), but for $\alpha = 17^\circ$. (E) As (D), but for $\alpha = 22^\circ$.

To assess the effects of the bioinspired wingtips on aerodynamic forces and corresponding flow fields, the aerodynamic properties of the baseline configuration are characterized first. As shown in Fig. 3A and B, stall occurs in stages for the baseline configuration. Lift gradually increases up to $\alpha = 17^\circ$, at which point, there is a sharp drop in lift paired with an abrupt increase in drag, characteristic of the onset of stall. Thus, the angle of attack regime of $\alpha < 17^\circ$ is referred to as the pre-stall regime. For $17^\circ \le \alpha \le 22^\circ$, there is a lift plateau until the onset of another drop in lift at $\alpha = 22^\circ$. These three stages can be explained by examining the average normalized spanwise vorticity, $\omega _z$, defined as the curl of the velocity field $\omega _z = (\frac{\partial v}{\partial x} - \frac{\partial u}{\partial y})$ and describing the local spinning motion of a fluid about the out-of-plane (here, *z*) axis. As expected, at $\alpha = 14^\circ$, which is in the pre-stall regime, the flow is attached for all captured PIV planes (Fig. 3C). At $\alpha = 17^\circ$, the flow progresses from being attached outboard at Plane 1, to slightly separated at the trailing edge at Plane 2, to fully separated at Plane 3 (Fig. 3D). Thus, the angle of attack regime of $17^\circ \le \alpha < 22^\circ$ is referred to as the partial stall regime. At $\alpha = 22^\circ$, the flow is separated for all PIV planes, indicating that stall is affecting most of the wing area (Fig. 3E). Therefore, the onset of full stall for the baseline configuration is considered to be at $\alpha = 22^\circ$.

**Fig. 4:**
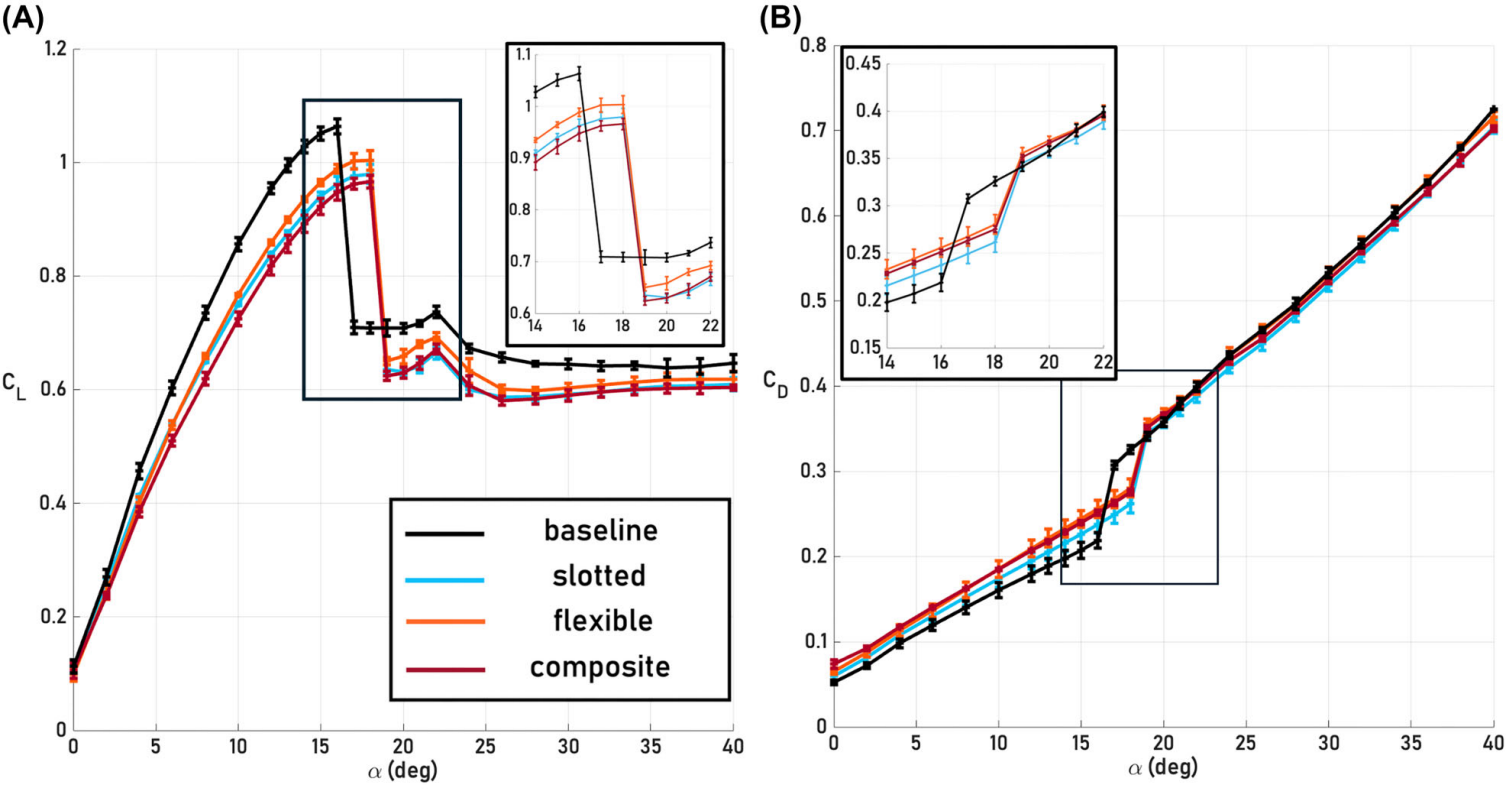
Lift and drag results for all configurations. (A) Mean $C_L$ as a function of $\alpha$ for the baseline, slotted, flexible, and composite configurations with vertical lines denoting the locations of pre-stall (14$^\circ$), partial stall (17$^\circ$), and full stall onset (22$^\circ$). Error bars here represent $\pm \sigma$. (B) Mean $C_D$ as a function of $\alpha$ for all configurations with the same vertical lines showing pre-, partial, and full stall onset locations, with error bars representing $\pm \sigma$.

**Fig. 5:**
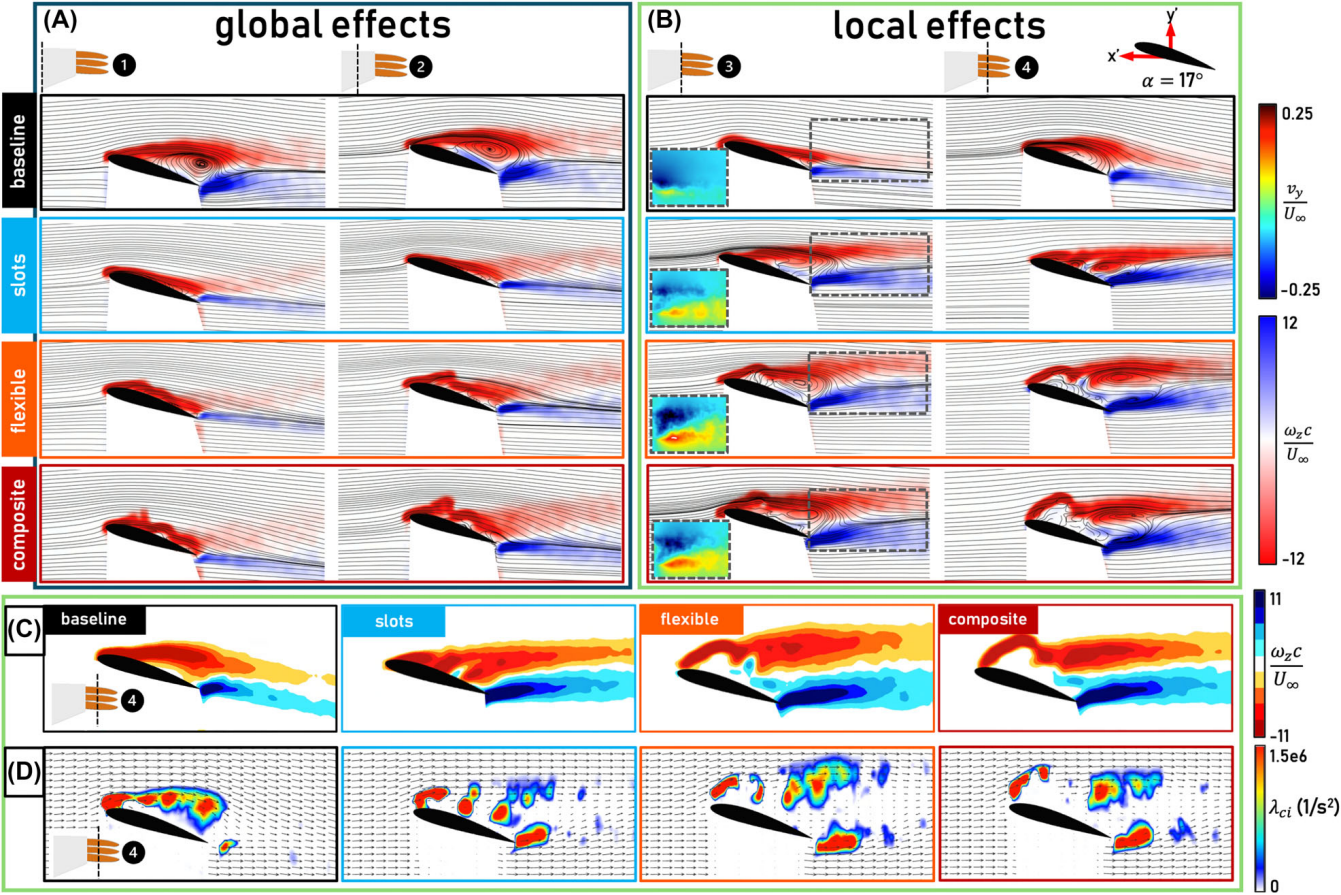
Flow fields showing the spanwise vorticity $\omega _z$ normalized by the mean chord *c* and freestream velocity $U_\infty$ for all configurations at the location of the baseline’s partial stall, $\alpha = 17^\circ$. Uniformly colored streamlines in (A) and (B) represent the velocity magnitude. (A) The two furthest inboard PIV planes, denoted by 1 and 2, which showcase the global effect of the wingtips. The baseline forms the top row, with the slotted, flexible, and composite configurations following below. (B) The two furthest outboard PIV planes, denoted by 3 and 4, showcase the local effect of the wingtips. Insets in Plane 3 exhibit the vertical velocity component $v_y$ normalized by $U_\infty$ in the wake region of each configuration. (C) Vorticity distribution heatmaps for, from left to right, the baseline, slotted, flexible, and composite configurations at the furthest outboard PIV plane, Plane 4. (D) Swirling strength $\lambda _{ci}$ for each configuration at Plane 4. Uniformly colored vectors represent the velocity magnitude.

**Fig. 6:**
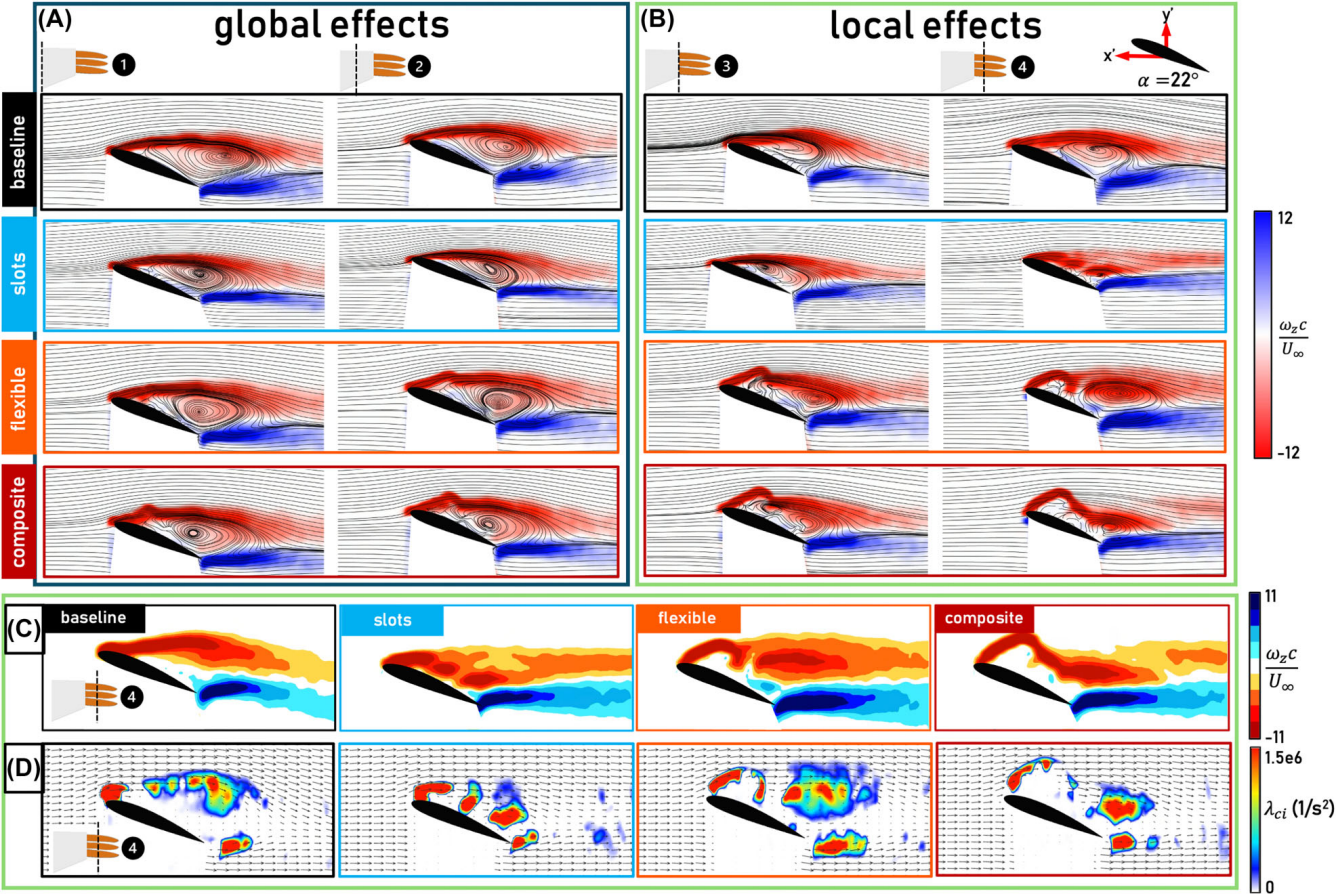
Flow fields showing $\omega _z$ normalized by *c* and $U_\infty$ for all configurations at the location of the onset of full stall, $\alpha = 22^\circ$. Uniformly colored streamlines in (A) and (B) represent the velocity magnitude. (A) The two furthest inboard PIV planes, denoted by 1 and 2, which showcase the global effect of the wingtips. The baseline forms the top row, with the slotted, flexible, and composite configurations following below. (B) The two furthest outboard PIV planes, denoted by 3 and 4, showcase the local effect of the wingtips. (C) Vorticity distribution heatmaps for, from left to right, the baseline, slotted, flexible, and composite configurations at the furthest outboard PIV plane, Plane 4. (D) $\lambda _{ci}$ for each configuration at Plane 4. Uniformly colored vectors represent the velocity magnitude.

Figure 4 shows that at $\alpha = 14^\circ$ all configurations remain in the pre-stall regime with the baseline having the highest lift. At $\alpha = 17^\circ$, however, where the baseline is in partial stall, the bioinspired wingtip configurations maintain lift production. This is explained by the flow fields shown in Fig. 5A, where flow separation is present in the baseline flow field at Planes 1 and 2, while the flow is fully attached for the bioinspired wingtip configurations at these inboard planes. Thus, in the partial stall regime, the bioinspired wingtip configurations effectively delay the spanwise stall propagation, as observed through a $2^\circ$ stall delay in the lift and drag signals (Fig. 4A and B). This delay in stall is evidence supporting the early hypothesis by Newman that slotted wingtips may act to control flow separation (Newman 1958). The fact that the bioinspired wingtip configurations affect the spanwise flow and overall lift distribution beyond their own spans, as evidenced by the flow fields at Planes 1 and 2, indicates that they have a global aerodynamic effect, defined as a measurable aerodynamic difference over the wing portion of the wing-wingtip system (i.e., Planes 1 and 2). Moreover, the flexible configuration has higher lift at several angles of attack compared to the other bioinspired wingtip configurations, both pre-stall and approaching full stall, while the composite and slotted configurations produce similar lift to one another throughout the $\alpha$ regime (Fig. 4A). The flexible configuration’s wake exhibits a stronger magnitude of negative normalized vertical velocity, or downwash (Plane 3 inset, Fig. 5 B) as a result of the large region of high magnitude vorticity shed from the significant upward bending of the leading edge and middle wingtips. This downwash explains its increased lift compared to the other bioinspired wingtip configurations. While the composite wingtips also incorporate bending flexibility, their downwash is lessened by the composite’s ability to twist, reducing each wingtip’s incidence angle and resulting in similar lift production to the slotted configuration. Extending the angle of attack regime beyond partial stall shows that all configurations experience the onset of full stall simultaneously at $\alpha = 22^\circ$, causing the global spanwise stall delay effect to diminish (Fig. 6A). The bioinspired wingtip configurations, while generally having lower lift than the baseline, generate similar or higher drag than the baseline across the angle of attack regime outside of the baseline’s partial stall. This indicates that the bioinspired wingtips do not reduce induced drag, unlike the findings of previous studies (Tucker 1995, 1993). Significant pressure drag is generated over the wingtip portion of the bioinspired wingtip configurations as showcased by Planes 3 and 4 in Figs. 5B and 6B which counteracts possible induced drag benefits. It is also difficult to isolate any induced drag effects without further spanwise and far-wake analyses, such as generating a downwash profile or studying the vertical and horizontal spreading of shed tip vorticity in a transverse plane as in KleinHeerenbrink et al. (2017).

Regardless of their effects on the lift and drag forces, all bioinspired wingtips exhibit local aerodynamic effects, defined as measurable differences in the flow field over the wingtip portion of the wing-wingtip system (i.e., Planes 3 and 4). A noticeable local effect is shown in Plane 3 during the partial stall regime. At Plane 3, the baseline’s flow is attached while the flow is separated for all of the bioinspired wingtip configurations (Figs. 5B, left column) and a similar trend is shown in Plane 4, where the baseline’s flow is partially attached, while the bioinspired wingtip configurations are fully separated (Figs. 5B, right column). Flow separation often results in a reduction of the pressure difference between the upper and lower surfaces of the wing, resulting in an overall reduced aerodynamic load. Thus, the flow fields at Planes 3 and 4 suggest that all of the bioinspired wingtip configurations reduce the aerodynamic load near the wingtip in the partial stall regime. However, the specific flow structures through which such reduction occurs differ across configurations (Figs. 5C and D). Figure 5C presents a vorticity distribution heatmap for each configuration, created by restricting the bounds of the averaged normalized spanwise vorticity scale and altering the color scheme. Figure 5D presents the swirling strength, $\lambda _{ci}$, for each configuration. Swirling strength further quantifies variations in the velocity field by isolating the swirling motion associated with a vortex from stretching or compression; in other words, swirling strength retains only rotational motion from a vortex. Separated peaks of high-intensity swirling strength suggest the presence of separated vortex cores (Zhou et al. 1999). From Figs. 5C and D, it is clear that the different DoFs incorporated by each bioinspired wingtip configuration shift the location of the separated shear layer and alter the distribution of vorticity. First, the location of the shear layer (Fig. 5C): the slotted configuration brings it closer to the wing surface, whereas the separated shear layer in the flexible configuration is farther from the wing surface due to bending deflections. The composite configuration’s effect on the shear layer location is an intermediate between the former two bioinspired wingtip configurations, as it combines the bending elasticity of the flexible wingtip configuration with twist, reducing the incidence angle of each wingtip. Regarding the vorticity distribution (Fig. 5D), unlike the baseline, where the vorticity and corresponding swirling strength remain cohesive over the wing surface, the bioinspired wingtip configurations exhibit isolated peaks of high-intensity swirling strength. The presence of isolated peaks of swirling strength is consistent with the results of KleinHeerenbrink et al. (2017) showcasing multiple vortex cores in the wake of a jackdaw’s slotted wingtips. The extent to which the vorticity is broken down into these isolated peaks is dependent on the incorporated DoF. The slotted configuration exhibits multiple peaks that appear behind the trailing edges of each wingtip. In contrast, for the flexible wingtip configuration, some of these multiple peaks coalesce into one cohesive region above the trailing edge wingtip, and again, the composite configuration’s flow field exhibits intermediate effects between the slotted and flexible wingtip configurations. Unlike the global aerodynamic effect, which diminishes at higher angles of attack, the local effects persist through both partial and full stall, as evidenced by the upward momentum in the wakes of the bioinspired configurations, which results in a reduction of lift, the difference in the location of the shear layer, and the separated peaks of high-intensity swirling strength shown in Figs. 6B–D. Thus, closely examining the flow fields near the wingtip reveals that the local aerodynamic effects of the bioinspired wingtips are the reduction of aerodynamic load over the wingtip portion of the wing, the alteration of the separated shear layer location, and the breakdown of tip vorticity.

**Fig. 7:**
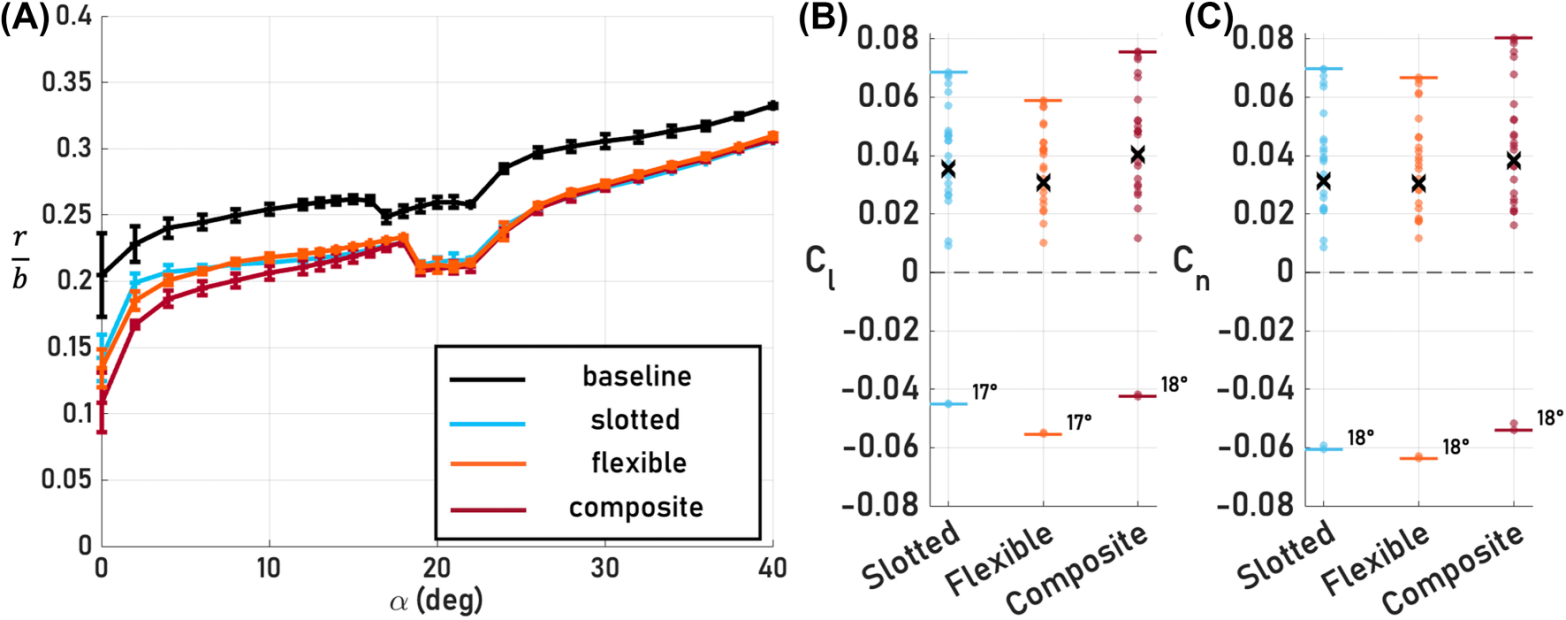
(A) Normalized rolling moment arm, defined as the rolling moment arm *r* normalized by the semi-span *b*, as a function of $\alpha$ for the baseline, slotted, flexible, and composite configurations with error bars representing $\pm \sigma$. A lower $r/b$ value indicates a more inboard location along the wing. (B) The rolling $C_l$ and (C) yawing $C_n$ moment coefficients resulting from subtracting the moments of a given bioinspired wingtip configuration from the baseline moments. The horizontal line at 0 is the reference line for the baseline; if points align with this line, there is no deviation from the baseline performance. The *x* indicates the mean value, while the whiskers show the range of values for various angles of attack $\alpha$.

The bioinspired wingtips’ global aerodynamic effect of tailoring the spanwise flow and separation propagation, and their local effect of reducing aerodynamic load over the wingtip portion of the wing, directly affect the spanwise lift distribution and, as a result, impact structural resiliency. This impact is reflected in the rolling moment arm, defined as the ratio of the rolling moment to the lift $\frac{M_L}{L}$, which serves as an approximation of where the center of lift acts along the wing span and indicates how the lift distribution varies across configurations as $\alpha$ changes. Figure 7A shows that the bioinspired wingtips shift the moment arm inboard relative to the baseline across the entire $\alpha$ regime. Moreover, across all angles of attack, the bioinspired wingtip configurations exhibit nearly identical moment arms, a consequence of their similar global aerodynamic effects. The consistent inboard shift of the rolling moment arm regardless of additional DoFs implies that changes in the lift distribution are not only due to an inward deflection of lift due to bending flexibility, as has been hypothesized previously (van Oorschot Klaassen 2020), and are not isolated to the outboard wing region; instead, it suggests a broader redistribution of lift toward the wing root due to the presence of slots. Additionally, shifting the resultant lift vector closer to the wing root reduces bending moment stresses by shortening the moment arm. For both natural and engineered fliers operating under high wing loading, this shift promotes greater structural resilience by enabling similar aerodynamic loading while reducing mechanical stress.

Finally, the bioinspired wingtips’ redistribution of the aerodynamic load over the wing is also linked to their modulation of lift and drag with respect to the baseline throughout the $\alpha$ regime (Figs. 4A and B), presenting a possibility for lateral control. An asymmetric redistribution of lift and drag can enable control authority by incorporating a differential configuration, where only one wing exhibits wingtip slotting. Such differential configurations are often exhibited in bird flight during banking and other maneuvers, where only one wingtip has pronounced slots. To characterize the potential control authority of the wingtips, the rolling $C_l$ and yawing $C_n$ moment coefficients are computed for each configuration, using:


(3) \begin{eqnarray*}
C_l = \frac{M_L}{\frac{1}{2}\rho U_\infty ^2 Sb},\,\,\,\,\,\,\,\,C_n = \frac{M_N}{\frac{1}{2}\rho U_\infty ^2 Sb},
\end{eqnarray*}

where a differential configuration is constructed by subtracting the mean values of the baseline configuration from those of each bioinspired wingtip configuration. The analysis indicates that the bioinspired wingtips are effective in creating rolling and yawing moments across the entire $\alpha$ regime (Fig. 7B and C). Moreover, during pre-stall, past partial, and full stall conditions, the moment sign is consistent with the only control direction reversal occurring at the partial stall angle of attack corresponding to each bioinspired wingtip (i.e., $\alpha = 17^\circ$ and $18^\circ$). Control reversal near partial stall occurs mainly because the lift difference between the baseline and the bioinspired wingtip configurations changes sign. In the pre-stall regime and during full stall, the baseline has higher lift, whereas in the partial stall regime, at the point of control reversal, the bioinspired wingtip configurations produce higher lift because of their ability to modulate flow separation propagation, thus reversing the sign of the difference and the resulting moment direction. The consistency of the directions of the rolling and yawing moments in the pre-stall regime is advantageous for control. For engineered vehicles, such consistency simplifies the design and implementation of control laws. Based on the force, moment, and flow field results, slotted wingtips can be used as flow-control devices near partial stall to mitigate flow separation. Conversely, away from stall, slotted wingtips can be deployed asymmetrically for roll and yaw control.

## Conclusion

This paper systematically investigates the properties of slotted wingtips to understand their aerodynamic mechanisms and associated flow physics. To that end, a simplified engineering analogy in the form of multiple wingtip configurations was designed to capture the individual DoFs seen in bird flight: slots, flexion, and twist. The results from force- and time-resolved PIV measurements reveal that slots, either alone or when coupled with flexion and twist, trigger a global aerodynamic effect in the form of delaying inboard stall propagation. Additionally, there are several local aerodynamic effects associated with the bioinspired wingtips: the reduction of aerodynamic load over the wingtip portion of the wing, the alteration of the separated shear layer location, and the breakdown of tip vorticity. The presence of slots, bending flexibility, and anisotropy-induced twist alters the structure of these local effects and the extent to which they are present, meaning that the resulting flow physics depend on the DoF exhibited. The global and local aerodynamic effects significantly impact lateral control and structural loading by changing the spanwise lift distribution, enabling consistent lateral control and reducing mechanical stress at the root for similar wing loading.

The flow fields showing the wingtips’ global effect of delaying spanwise stall propagation constitute the first evidence supporting the initial hypotheses made by Graham and Newman that slotted wingtips may act as flow separation control devices (Graham 1932; Newman 1958). Additionally, the local effect of the breakdown of tip vorticity is consistent with results presented by Tucker and KleinHeerenbrink showing bird wings with slotted wingtips spreading the tip vortex horizontally and vertically (Tucker 1993; KleinHeerenbrink et al. 2017). However, the results of this study do not show that there is an induced drag benefit associated with slotted wingtips, in contrast to previous work (Spillman 1978; Tucker 1995). Induced drag is better characterized using spanwise flow fields in the wake region, where downwash is present, and thus requires further analysis than what is presented in this study. Additionally, although the results show local differences in the flow physics between the bioinspired wingtip configurations, these differences do not appear to significantly affect the global aerodynamic forces beyond those discussed for the flexible configuration in comparison to the other bioinspired wingtip configurations. This may be attributed to differences between biological feathers and the engineered wingtips used in this study. For example, the upward deflection of barn owl primary feathers is much less than that of Harris’s hawks. However, given the limited data on the elasticity of Harris’s hawks’ feathers, it was assumed that basing the bioinspired wingtips on the barn owl data would at least result in an elasticity regime that is relevant to biological feathers.

Nonetheless, the results of this study expand upon the current understanding of the functional morphology of slotted wingtips by providing evidence that slotted wingtips significantly impact spanwise flow and employ multiple aerodynamic mechanisms, including some that are DoF-dependent. Specifically, these mechanisms are: spanwise flow separation control, reducing aerodynamic load at the wingtip, altering the location of the shear layer, and breaking down tip vorticity. Together, these mechanisms result in global aerodynamic changes, such as stall delay, control authority, and structural resiliency, demonstrating the benefits of a simplified bioinspired flow-control device. Based on these results, an engineering system incorporating slotted wingtips should only feature slots and deploy them in an open position during stall due to their global effect, or asymmetrically for lateral control capability away from stall. These suggested conditions in which to feature wingtip slots align closely with initial observations by Graham and Newman (Graham 1932; Newman 1958) of birds with slotted wingtips switching from open and closed slot configurations during flight, lending credibility to the use of carefully designed engineered analogies to provide insights into the role of slotted wingtips in biology and inform the design of bioinspired flow-control devices for improved engineered fliers.

## Author contributions

Hannah Wiswell (Data curation, Formal analysis, Methodology, Software, Validation, Visualization, Writing – original draft, Writing – review & editing), Aimy Wissa (Conceptualization, Funding acquisition, Project administration, Resources, Supervision, Writing – review & editing)

## Supplementary Material

icag036_Supplemental_Files

## Data Availability

The data underlying this article will be shared upon reasonable request to the corresponding author.

## Notes

From the symposium “Dinosaurs to Drones: Comparative Approaches to Understanding Flight” presented at the annual meeting of the Society for Integrative and Comparative Biology, January 3–7th, 2026.
