# Supplementary figures and images for: Aerodynamic Mechanisms and Flow Physics of Bioinspired Slotted Wingtips
Source: Integr Comp Biol. 2026 May 5;66:icag036. doi: 10.1093/icb/icag036 (PMC13176839; doi:10.1093/icb/icag036)

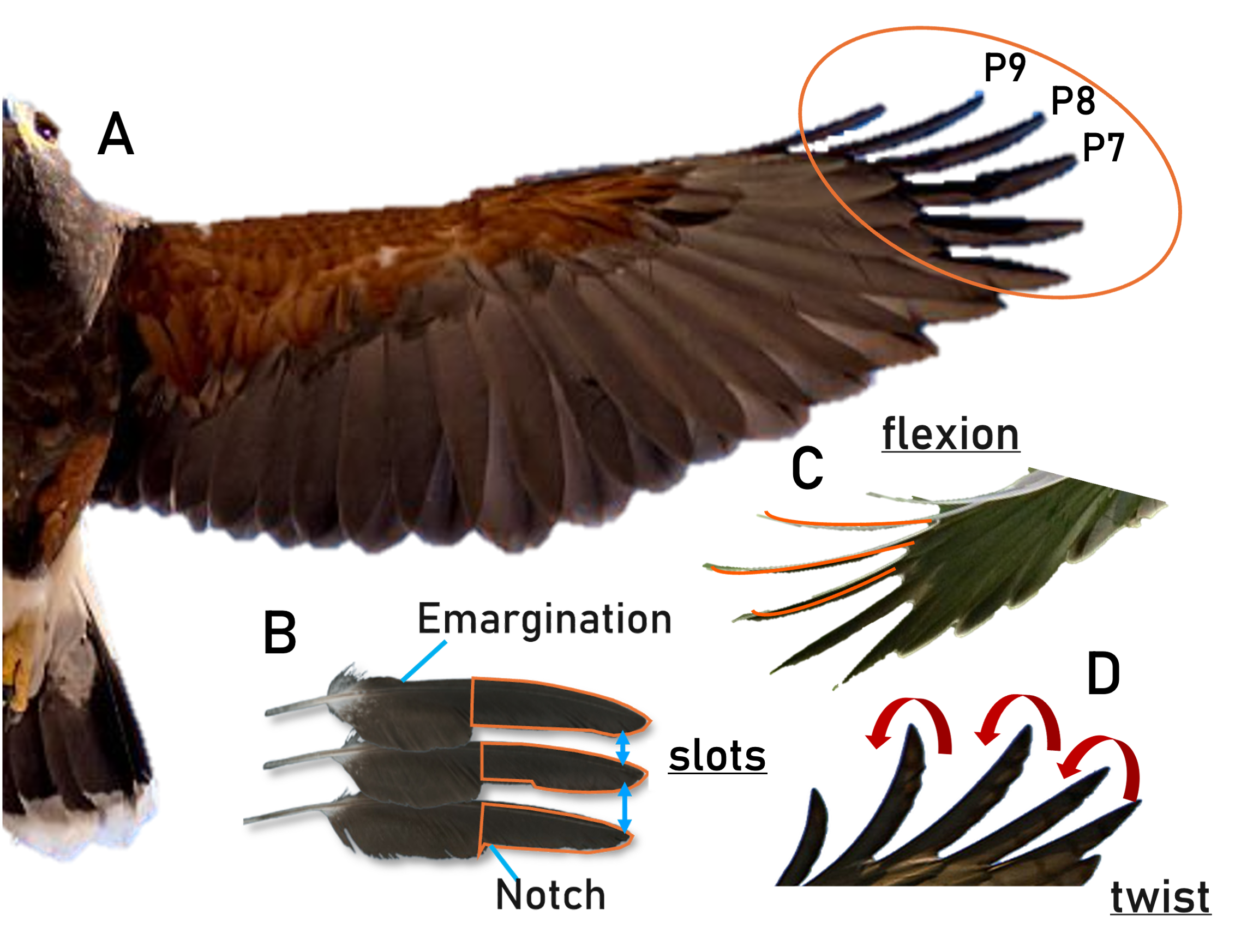

Supplement: icag036_Supplemental_Files [file icag036_supplemental_files.zip › icb-2026-0035-File004.png]

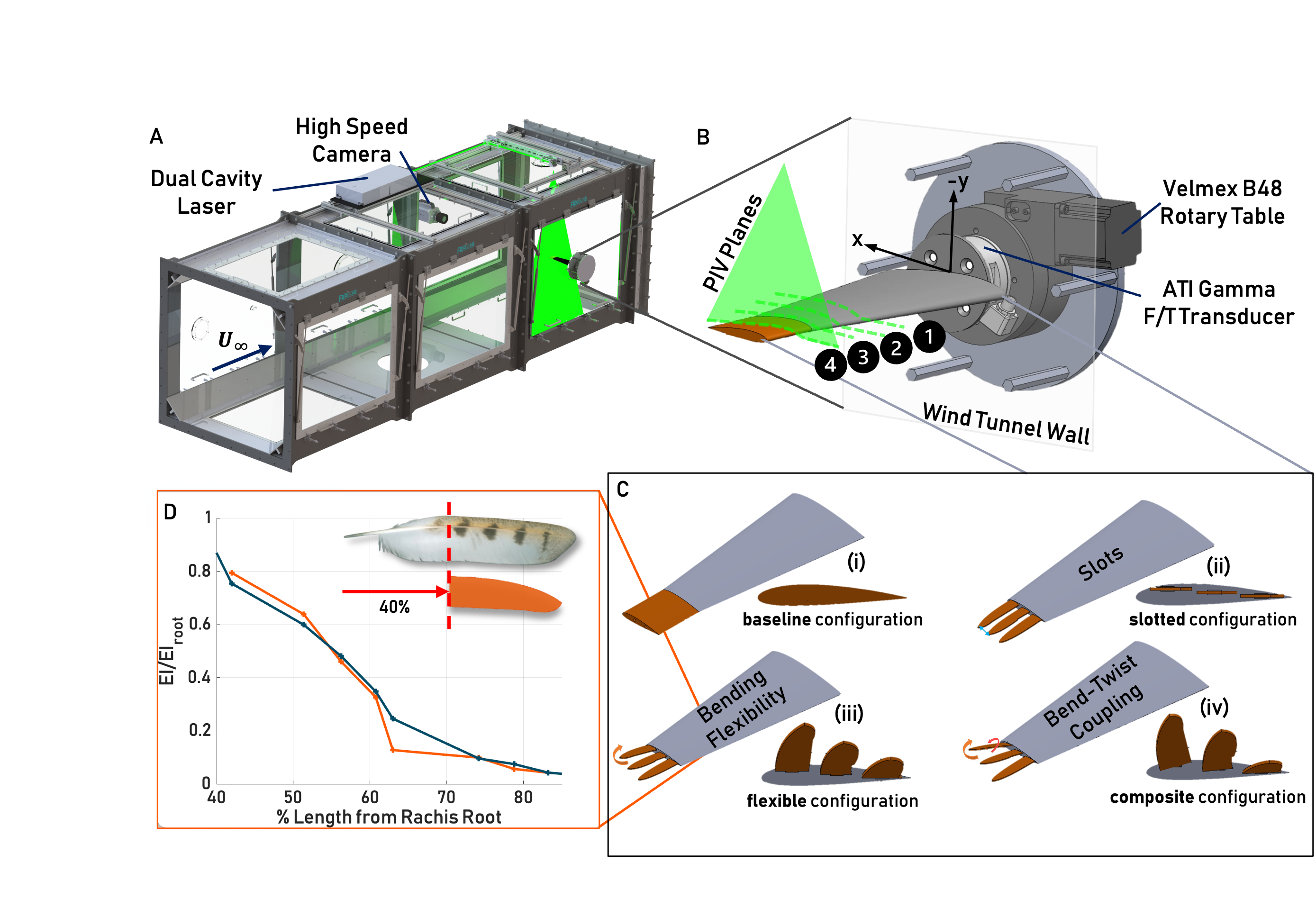

Supplement: icag036_Supplemental_Files [file icag036_supplemental_files.zip › icb-2026-0035-File005.png]

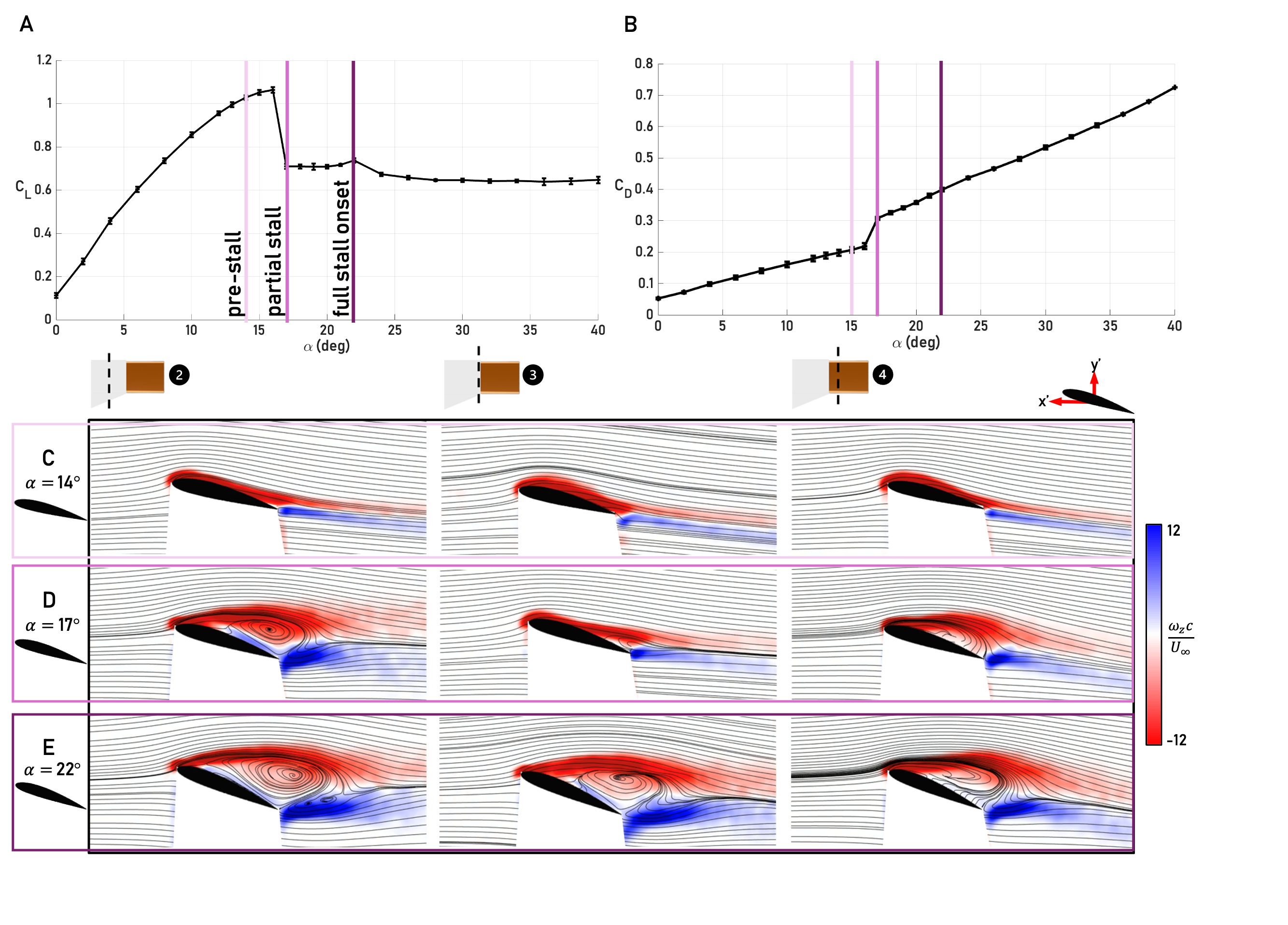

Supplement: icag036_Supplemental_Files [file icag036_supplemental_files.zip › icb-2026-0035-File006.png]

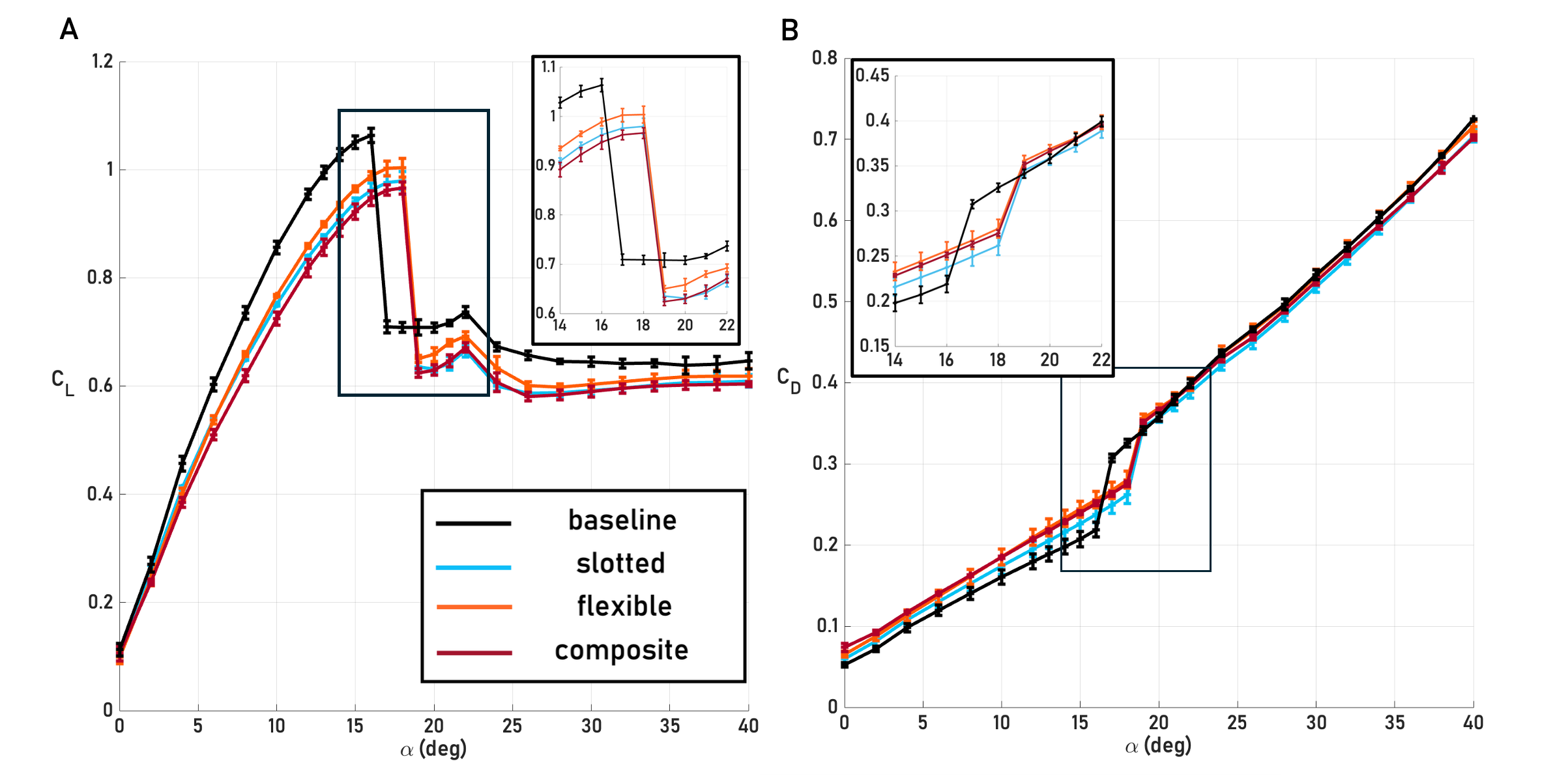

Supplement: icag036_Supplemental_Files [file icag036_supplemental_files.zip › icb-2026-0035-File007.png]

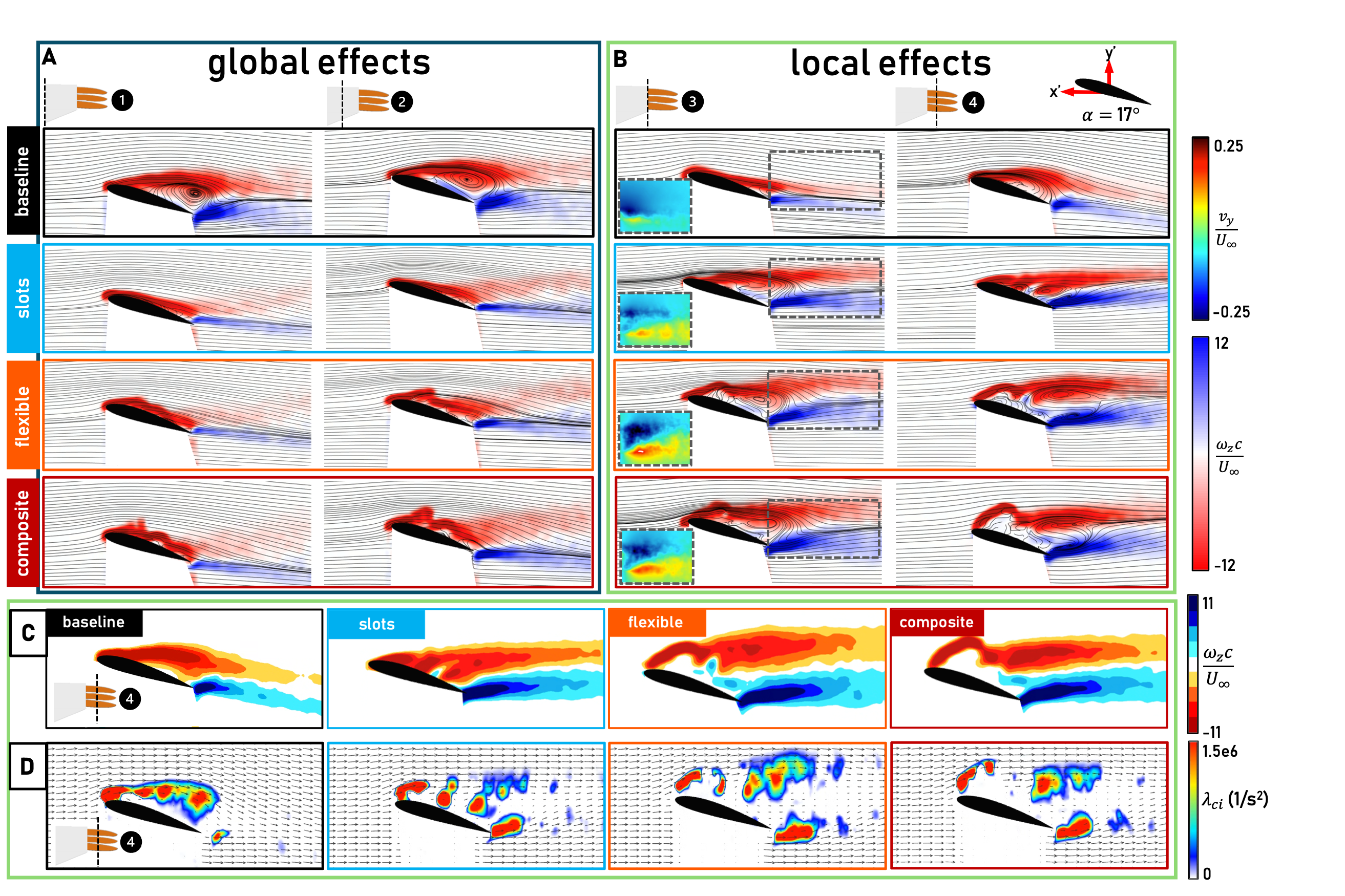

Supplement: icag036_Supplemental_Files [file icag036_supplemental_files.zip › icb-2026-0035-File008.png]

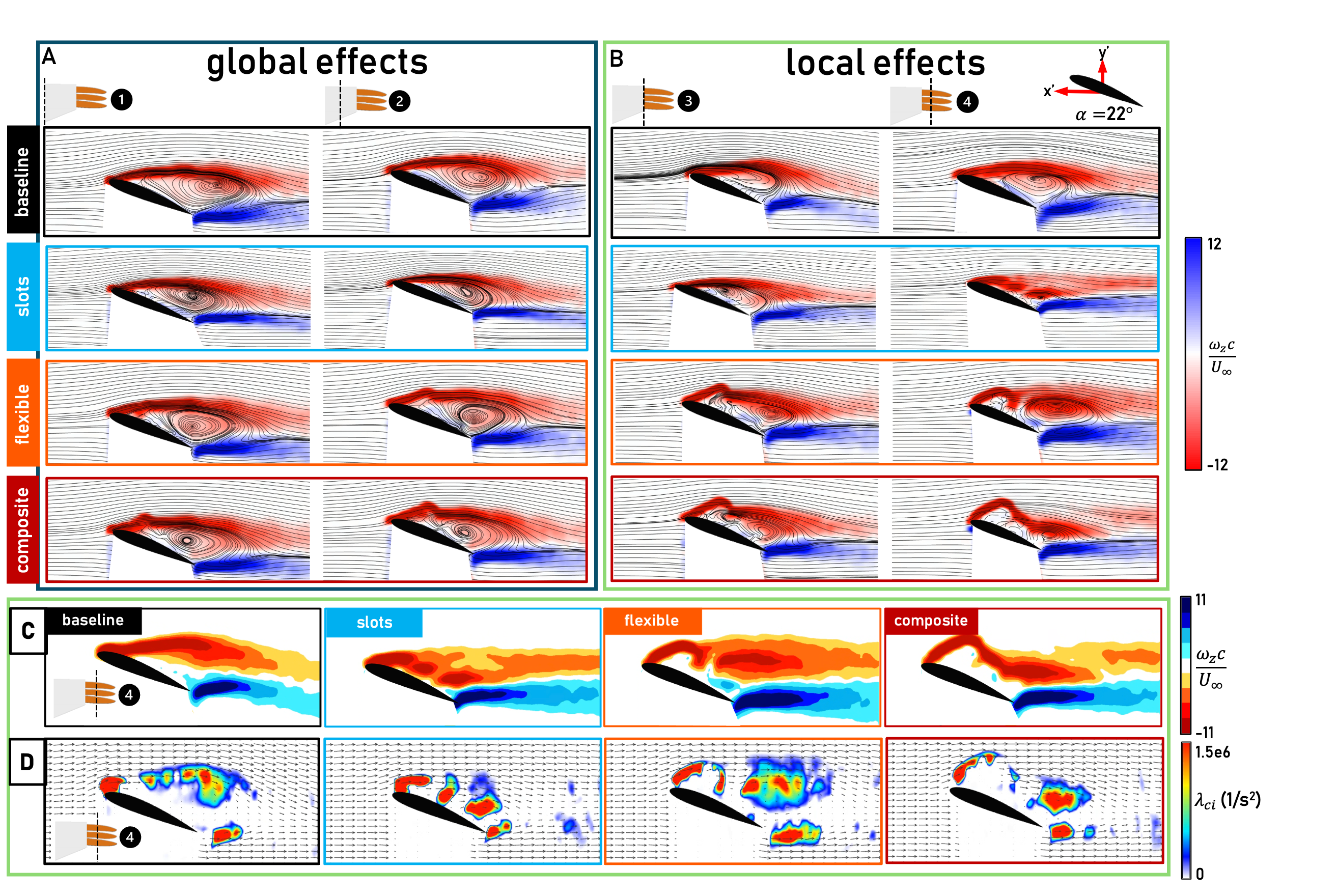

Supplement: icag036_Supplemental_Files [file icag036_supplemental_files.zip › icb-2026-0035-File009.png]

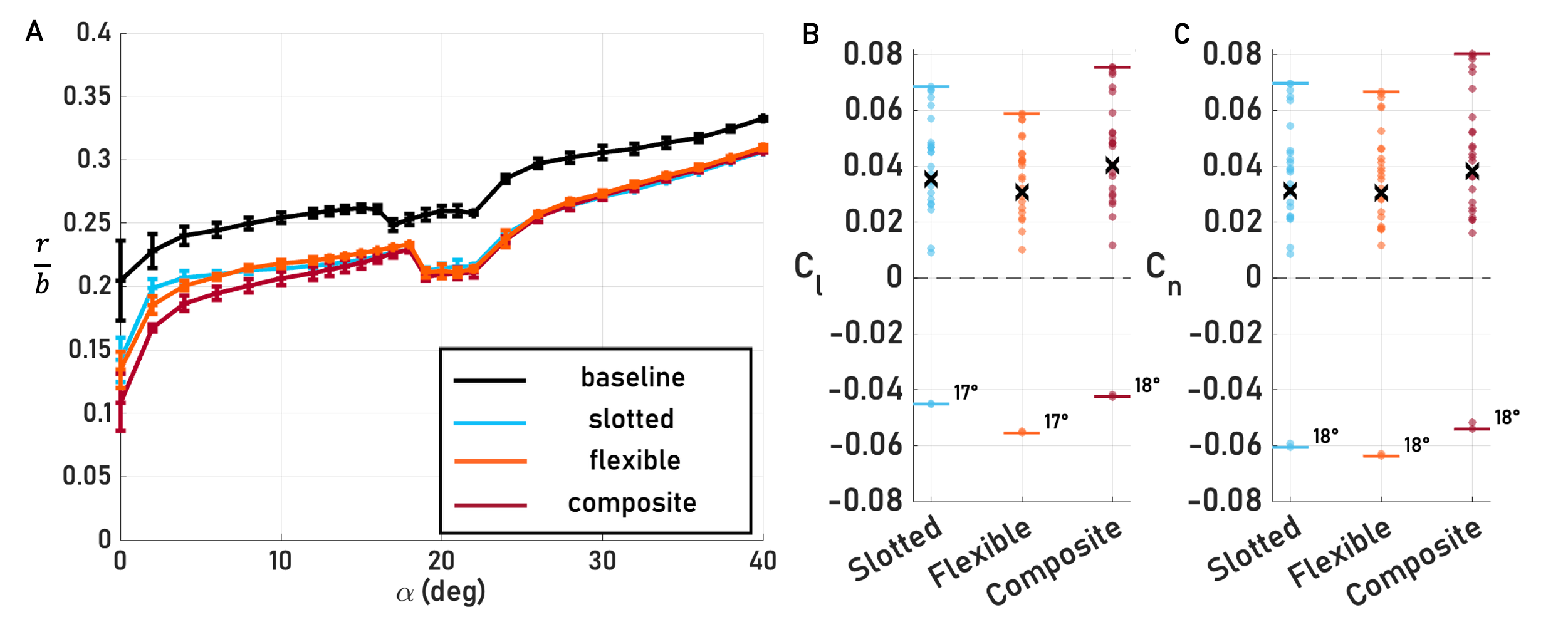

Supplement: icag036_Supplemental_Files [file icag036_supplemental_files.zip › icb-2026-0035-File010.png]
